# Temporal dynamics of lymphocytes in prostate cancer patients treated with proton therapy

**DOI:** 10.3389/fonc.2025.1470876

**Published:** 2025-04-16

**Authors:** Sarah Salih Al-Hamami, Samuel Kurucz, Vladimír Vondráček, Vladimír Pekar, Michal Andrlík, Kateřina Dědečková, Iva Benešová, Alexandra Haas, Barbora Ondrová, Andrea Pasztorová, Pavel Vítek, Jiří Kubeš

**Affiliations:** ^1^ Department of Radiation Oncology, Proton Therapy Center Czech, Prague, Czechia; ^2^ Department of Medical Physics, Proton Therapy Center Czech, Prague, Czechia; ^3^ Department of Immunology, Second Faculty of Medicine, Charles University and University Hospital Motol, Prague, Czechia

**Keywords:** prostate cancer, proton therapy, radiation oncology, hypofractionation, immunostimulation, lymphopenia

## Abstract

Radiotherapy can be both immunosuppressive and immunostimulatory. Radiation-induced lymphopenia (RIL) is an ongoing challenge in cancer treatment. We investigated weekly changes in the absolute lymphocyte count (ALC) during proton radiotherapy, evaluating the effects of different dosage, fractionation schedules, and pelvic node irradiation (PNI). Prostate cancer patients were prospectively chosen for this study, due to their relatively homogenous treatment plans. Treatment protocols were categorized into three groups: Group A (n=52) received 36.25 Gy/5-fractions, Group B (n=60) underwent 63 Gy/21-fractions and group C (n=69) received 63 Gy/21-fractions plus PNI. To account for individual characteristic differences, a new categorization method was made, according to the change in ALC relative to the baseline. Lymphopenia (ALC < 1000 K/μL) developed in 8%, 17% and 84% of patients in groups A, B, and C, respectively. An initial increase in ALC occurred in 44%, 47% and 28% of groups A, B and C, respectively, and declined with proceeding fractions. Patients with PNI had the most pronounced reduction in their ALC relative to the baseline. Increased dosage and fractionation led to a higher incidence of lymphopenia. Understanding which factors influence ALC in particle therapy is vital for leveraging the immune-enhancing effects of radiotherapy, while minimising its immunosuppressive impacts.

## 1 Introduction

Radiotherapy, a fundamental part of cancer treatment, has evolved significantly over the years leading to improved cancer control and reduced treatment-related toxicities. Amidst these advancements, the impact of radiotherapy on the immune system has emerged as an important frontier of research. A literature review revealed that some degree of immunosuppression occurs in 40-70% of patients with solid tumours treated with radiotherapy, independent of steroid use or concurrent chemotherapy (1). This immunosuppression manifests primarily as a reduction in lymphocytes, which with an LD_50_ of just 2 Gy, are known to be the most radiosensitive cells in the peripheral blood (2).

In the last decade, concerns around radiation-induced lymphopenia (RIL) have grown, particularly in conjunction with the increasing mainstream use of immunotherapy. Research investigating the relevance of RIL has surged, and a meta-analysis covering 20 studies confirmed a notable association between RIL and overall survival: patients with lymphopenia of grade ≥ 3 faced a 65% increased risk of death compared to those with grades 0-2, with an additional risk of 50% for those with grade 4 compared to grades 0-3 (3).

To build appropriate measures aimed at reducing RIL, we must first understand its complex, multifactorial pathophysiology. A spectrum of factors influence the degree of RIL, including varying dosimetric parameters, radiation modalities, fractionation schedules and dosages (4).Radiation can cause direct damage to circulating lymphocytes and lymphoid-rich organs such as the bone marrow, lymph nodes, spleen, and thymus, resulting in RIL (1, 5). Conversely, radiotherapy can stimulate the immune system (6). Radiotherapy induces DNA damage in cancer cells, which gives rise to neoantigens that could be targeted by immune cells previously unresponsive to these cancer cells. Moreover, RT can amplify immunogenic cell death in cancer cells, leading to an increased release of tumour antigens and damage-associated molecular patterns (DAMPs) (7). This process triggers anti-tumour immunity by activating dendritic cells, enhancing antigen presentation, and promoting the expression of proinflammatory cytokines (such as TNF-α, interferon-α, β, and γ) as well as chemokines that attract immune cells (7, 8). These mechanisms may ultimately result in better tumour elimination by CD8^+^ T cells, NK cells and macrophages (9). This situation has appropriately been termed an *in-situ* vaccine, as it can lead to a systemic anti-tumour response (10).

However, this mechanism of activating the immune system can paradoxically trigger immunosuppressive pathways, such as the IFN-γ mediated upregulation of immune checkpoint molecules (e.g., PD-L1) (11). Moreover, radiotherapy may increase the amount of circulating and tumour-infiltrating regulatory T cells (Tregs), myeloid-derived suppressor cells (MDSC) and tumour-associated macrophages (TAMs). These cells suppress anti-tumour immunity at multiple levels. While this may limit the immunostimulatory effects of radiotherapy alone, combining it with immunotherapy, such as a PD-L1 inhibitor, could lead to a synergistic effect (12).

The composition and state of the tumour immune microenvironment (TIME) might be a decisive factor regarding the outcome of radiotherapy on anti-tumour immunity. The TIME of prostate cancer is considered immunologically cold, characterised by relatively low immune cell infiltration compared to tumours such as melanoma or renal cell carcinoma, which exhibit high immune cell infiltration (13). The immune cells present are often anergic or immunosuppressive, and there is frequently a loss or reduction in the expression of MHC molecules. Consequently, radiotherapy may serve as an effective tool for transforming these immune cold tumours into hot ones (14).

In this study, we present our experience with radiation-induced lymphocyte count changes in patients undergoing proton therapy for prostate cancer. To minimise the impact of individual patient variables, we selected prostate cancer patients as they represent a more homogeneous cohort in terms of treatment planning and clinical characteristics. We investigated the change in lymphocyte count under different doses and fractionation schemes. Additionally, we sought to investigate the impact of pelvic lymph node irradiation (PNI) on the incidence and severity of lymphopenia. We hope that our research contributes to the current understanding of how the immune system responds to radiation therapy.

## 2 Materials and methods

### 2.1 Patient population

We conducted a prospective cohort study on patients with histologically confirmed, localised, or locally advanced prostate cancer treated at a single proton therapy centre between July 2022 and September 2023. Blood work of patients was collected and analysed along with their clinical status and dosimetric data. A separate analysis was done to determine what could be considered a sufficient endpoint. Patients that had their last blood sample taken after completing less than 86% of the treatment were excluded. The inclusion and exclusion criteria are detailed in **Table 1**.

**Table 1:** Inclusion and exclusion criteria for the cohort of prostate patients selected for evaluation of their lymphocyte count dynamics while undergoing proton therapy.

Inclusion criteria	Exclusion criteria
• Histologically confirmed prostate cancer	• Radiological evidence of distant metastases (M1)
• Indication for radical treatment with proton therapy	• History of radical prostatectomy
• Scheduled for treatment with either 5 or 21 fractions	• Incomplete blood results i.e. missing time points
• Baseline ALC taken within 7 weeks of starting radiotherapy	• Current or recent (within 6 months) use of immune-modulating agents such as corticosteroids or immunotherapy
• Weekly complete blood counts blood tests during treatment	• Patients with autoimmune diseases, haematological malignancies, primary or secondary/acquired immunodeficiencies, chronic infections, severe anaemia or cytopenias or genetic disorders affecting immunity
• Completed full course of proton therapy	• Prior radiotherapy in the pelvic region

**Figure 1:**
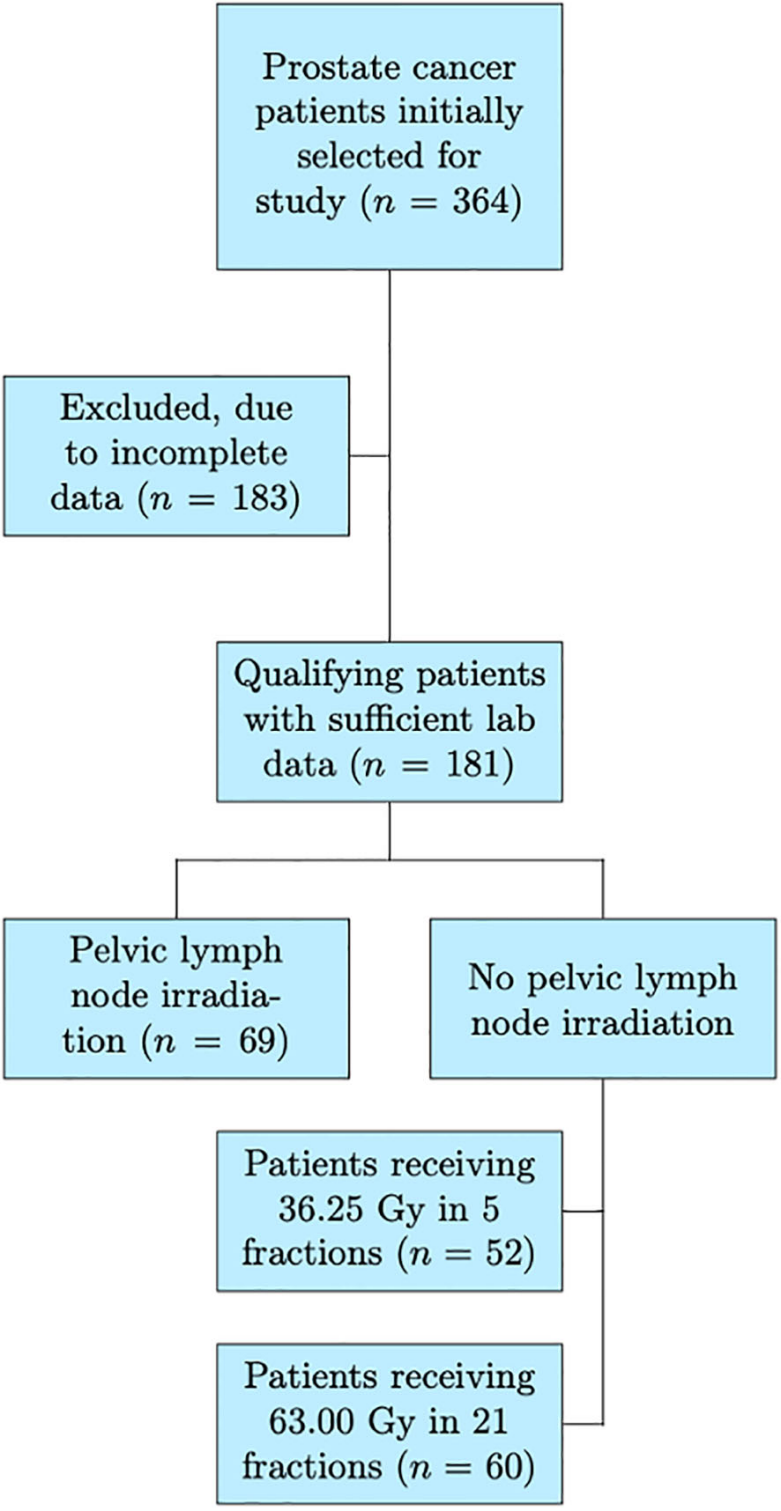
A flowchart illustrating the study’s patient selection process, the final cohort included in the analysis, and the three subcohorts. Numbers at each step represent the counts of patients included or excluded.

Patients were divided into three groups: those in the ultra-hypofractionated regime receiving 5 fractions with a total dose of 36.25 Gy (Group A), those undergoing a regime of 63 Gy across 21 fractions (Group B), and patients receiving 63 Gy in 21 fractions with additional pelvic lymph node irradiation (Group C). Patients provided written informed consent, all data has been anonymised, and the institutional ethics committee approved this study (ID: 2023015). The patient selection process can be found in **Figure 1**.

### 2.2 Treatment methods

All patients received definitive proton radiotherapy using the pencil beam scanning technique, with treatment planning performed on the Raystation**^®^** treatment planning system (RaySearch Laboratories AB). Planning procedures, contouring methods, treatment planning as well as target volumes are described in a previous article (50). The prescribed dose to the prostate for patients without PNI was 36.25 Gy in 5 fractions, or 63 Gy in 21 fractions. The dose for patients with PNI was 63 Gy in 21 fractions to the prostate gland, with a concomitant 48.3 Gy to the lymph nodes and seminal vesicles. Treatment protocols were selected for patients according to institutional guidelines, depending on tumour stage, risk group, and patient-specific factors.

### 2.3 Blood analysis

Peripheral blood was collected at three (Group A) or five (Groups B, C) different time points; before treatment (baseline), during treatment at weekly intervals, and at the end of the treatment. The peripheral blood was collected into EDTA tubes and immediately transferred to an independent laboratory under controlled conditions at room temperature, to ensure sample integrity. At the laboratory, absolute lymphocyte counts (ALC) were assessed using fluorescent flow cytometry, using a semi-conductor laser with hydrodynamic focusing in dedicated channels, on the XN-1000™ Automated Hematology Analyzer (Sysmex, Kobe, Japan). The results were generated as the absolute number of lymphocytes per litre of blood. The ALC, derived from a complete blood count analysis, represents the total lymphocyte population, including B cells, T cells, and NK cells.

### 2.4 Definition of radiation-induced lymphopenia in absolute and relative terms

RIL was defined as a decrease in the peripheral ALC to below the lower limit of the acceptable range (ALC < 1,000 K/μl), after exposure to ionising radiation. The severity of lymphopenia was graded according to the National Cancer Institute’s Common Terminology Criteria for Adverse Events (CTCAE) v5.0 as follows: grade 1 (G1) – ALC 999 K/μl to 800 K/μl; grade 2 (G2) – ALC 799 K/μl to 500 K/μl; grade 3 (G3) – ALC 499 K/μl to 200 K/μl; grade 4 (G4) – ALC < 200 K/μl.

Grading of lymphopenia in this way does not take into account the decrease of ALC relative to the baseline. Therefore, we developed a categorisation for the relative change across six levels, defined as follows:

Level + (L+) – ALC increase of more than 5% relative to the baseline;
Level 0 (L0) – ALC increase of less than 5% and decrease of less than -5% relative to the baseline;
Level 1 (L1) – ALC decrease between 5–25% relative to the baseline;
Level 2 (L2) – ALC decrease between 25–50% relative to the baseline;
Level 3 (L3) – ALC decrease between 50–75% relative to the baseline;
Level 4 (L4) – ALC decrease of more than 75% relative to the baseline.

A buffer zone of +/- 5% was used to separate L+ and L1, to account for physiological fluctuations in ALC as well as any possible laboratory inaccuracies in the blood analysis.

### 2.5 Statistical analysis

Descriptive statistics were used to summarise the patient population, treatment characteristics, and lymphopenia outcomes, between groups B and C. Group A was excluded from the univariate (UVA) and multivariate analyses (MVA) due to its distinct treatment protocol, ensuring comparability and consistency among the groups studied. Patient variables included in the UVA were: age, Gleason score according to the International Society of Urological Pathology (ISUP) guidelines, initial prostate-specific antigen (PSA), baseline lymphocyte count, hypertension (yes/no) and diabetes mellitus (DM) (yes/no). Treatment variables included use of antihormonal therapy (yes/no), PNI (yes/no), and cumulative dose at nadir, the first in-treatment, and the last sample, for relevant outcome variables. The planning treatment volume (PTV) was not included as there is a high degree of collinearity with the PNI variable. Outcome variables were defined as ALC < 500 K/μl (high-grade lymphopenia), ALC < 1000 K/μl (any grade of lymphopenia), relative change in ALC compared to the baseline at the first week and at the end-of-treatment sample. The normality of continuous variables was evaluated using Shapiro-Wilk test where applicable and visually using histograms if not.

Univariate linear regression (OLS method) was used to identify the predictor variables that would go on to be included in the multivariate linear regression. The UVA cut-off value of α = 0.20 for p-values was considered significant enough to be evaluated further. Multivariate linear regression (OLS method) was then performed to identify the significance of the predictors. A cut-off value of α = 0.05 for p-values was considered statistically significant. Every p-value in this study was two-tailed. All statistical analyses were conducted using python programming language.

The relative changes in ALC by the end of the first week were further analysed to better assess the potential immunostimulatory effects observed in some patients. Patients from groups B and C that had their first in-treatment sample at cumulative doses of exactly 6 or 9 Gy were analysed. The ratio of patients in L+ (versus L1-L4) between the groups was verified using a chi-squared test, where a cut-off value of α = 0.05 for p-values was considered statistically significant. Patients in the buffer zone were not included in this analysis. The chi-squared test was also used for evaluating the statistical significance of other proportional comparisons, where applicable.

## 3 Results

### 3.1 Patient characteristics

A total of 181 patients with prostate cancer treated with proton radiotherapy with curative intent were evaluated in this study. Fifty-two patients received 36.25 Gy and no PNI (Group A; n = 52), 60 patients underwent 63 Gy and no PNI (Group B; n = 60) and 69 patients received 63 Gy with PNI (Group C; n = 69). The median age for the entire cohort was 70 years (range: 45–84 years). The total planning treatment volume (PTV) varied substantially between the PNI and non-PNI groups. Groups A and B had median PTV values of 118.4 cm^3^ (67.3–180.2 cm^3^) and 171.6 cm^3^ (50.1–355.4 cm^3^), respectively, while the median PTV for group C was much larger, at 868 cm^3^ (595–1599.8 cm^3^). Detailed patient characteristics can be found in **Supplementary Table S1** in the **Supplementary Material**.

### 3.2 Baseline and sampling details

**Figure 2:**
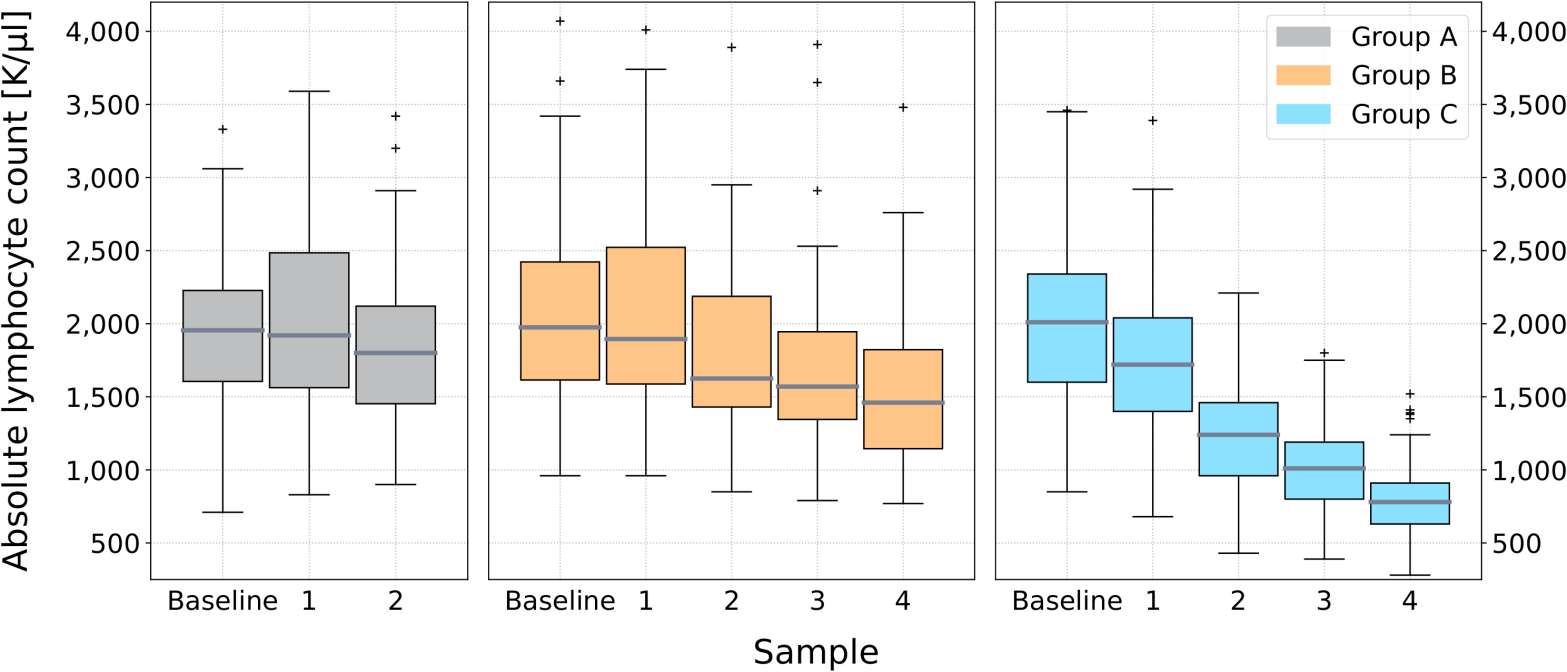
Box plots of the absolute lymphocyte count throughout treatment for groups A, B and C. Absolute lymphocyte count measurements are shown at baseline (prior to the start of radiotherapy) and at regular intervals during treatment. The boxes represent the interquartile range (IQR), the horizontal line within each box indicates the median, and the whiskers extend to the minimum and maximum values. Outliers are shown as individual points.

The ALC was recorded and analysed for all three groups throughout the course of treatment (**Figure 2**). The median baseline value for the entire cohort was 1.97 K/μl (0.71-4.74 K/μl). Consecutive samples were taken after the baseline, at an average dose of: Sample 1– Group A: 12.13 Gy (s.d.: 3.99 Gy), Group B: 9.35 Gy (s.d.: 3.99 Gy), Group C: high dose – 10.48 Gy (s.d.: 4.50 Gy), low dose – 8.03 Gy (s.d.: 3.45 Gy); Sample 2– Group A: 33.46 Gy (s.d.: 3.56) (end-of-treatment), Group B: 23.85 Gy (s.d.: 4.33 Gy), Group C: high dose –24.65 Gy (s.d.: 5.22 Gy), low dose – 18.90 (s.d.: 4.0); Sample 3– Group B: 38.60 (s.d.: 5.20 Gy) Gy, Group C: high dose – 39.17 Gy (s.d.: 5.44 Gy), low dose: 30.03 (s.d.: 4.17 Gy); Sample 4 (end-of-treatment) – Group B: 59.30 Gy (s.d.: 3.42 Gy), Group C: high dose – 59.78 Gy (s.d.: 3.18 Gy), low dose – 45.83 Gy (s.d.: 2.44). We aimed to standardise blood sampling as much as possible; however, factors such as differing treatment start days throughout the week, weekends, pauses in treatment, and other variables resulted in some variation between patients. Details on the total cumulative dose, the number of fractions, and the days after starting radiotherapy for each sample are provided in **Supplementary Table S2** in the **Supplementary Material**.

### 3.3 Changes in lymphocyte count during treatment

As seen in **Figure 2**, there is a discernible downward trend of the median ALC with progression of treatment across all three sub-cohorts. Groups A and B demonstrate a gradual decrease, whereas group C exhibits a steeper decline. At the first in-treatment sample, the median ALC was 1.92 (0.83-3.59) K/μl, 1.90 (0.96-4.37) K/μl and 1.72 (0.68-3.39) K/μl for groups A, B and C, respectively. The second sample provided the end-of-treatment ALC value for group A, with a median value of 1.80 (0.90-3.42) K/μl, resulting in a 6.3% sample-to-sample decline. The largest sample-to-sample change for groups B and C occurred between samples 1 and 2. The ALC declined by 14.2% [ALC: 1.63 (0.85-3.89) K/μl] for group B, and 27.9% [ALC: 1.24 (0.43-2.21) K/μl] for group C. At samples 3 and 4, there was a consecutive decline of 3.7% [ALC: 1.57 (0.79-3.91) K/μl], then 7.0% [ALC: 1.46 (0.77-3.48) K/μl] for group B, suggesting a plateau occurring around 23.9 Gy. Group C continued to decline steeply, by 18.5% at sample 3 [ALC: 1.01 (0.39-1.80) K/μl], then 22.8% [ALC: 0.78 (0.28-1.52) K/μl] by the end of treatment.

### 3.4 Lymphopenia grade reached at nadir

**Figure 3:**
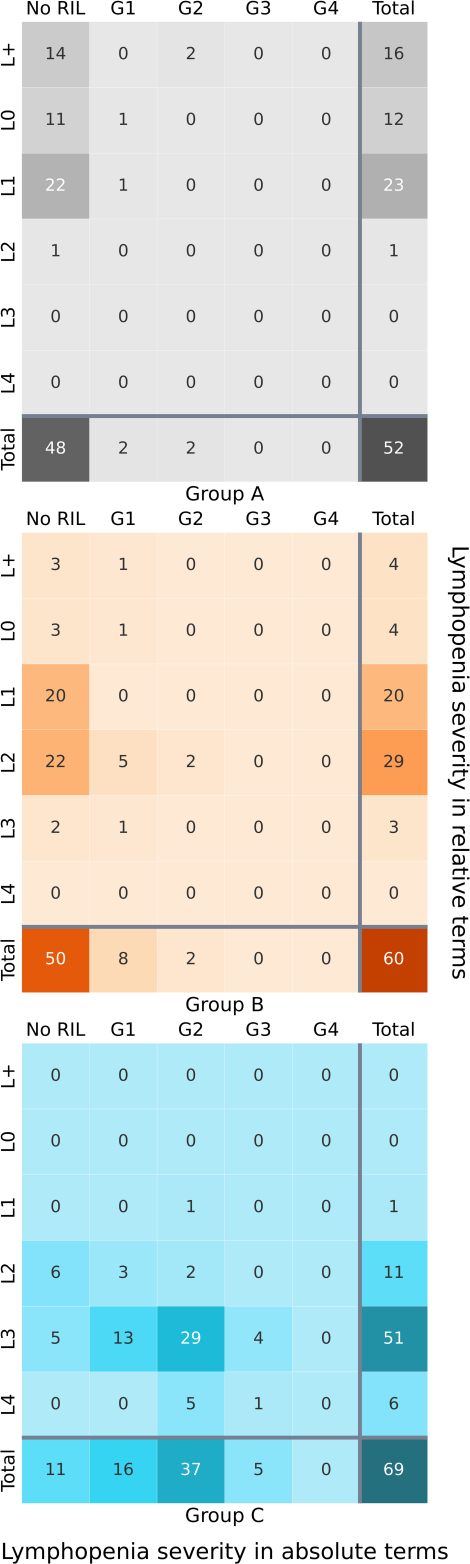
Heatmap of cross-categorisation between nadir absolute lymphocyte count grade (lowest count during treatment) and the relative decline in absolute lymphocyte count by the end of treatment compared to the baseline, for groups A, B and C. Darker colours indicate higher frequencies of patients within specific categories.

The majority of group A (92.3%) did not develop acute RIL (**Figure 3**). Two patients developed G1 RIL (3.8%) and two reached G2 at nadir (3.8%). In group B, 83.3% of patients did not experience RIL. There were slightly more patients in G1 (13.3%), and two patients in G2 (3.3%). Only 15.9% of patients with PNI (group C) did not develop RIL. The majority of patients resulted in G2 RIL at nadir (53.6%). Sixteen patients (23.2%) had G1, and five patients (7.2%) reached a nadir of G3 (ALC < 499 K/μl). No patients from any group developed G4 RIL (ALC < 200 K/μl).

### 3.5 Change in lymphocyte count relative to the baseline

It is important to consider that grading of lymphopenia according to the ALC at nadir, does not accommodate for individual characteristics of patients that may affect the state of their baseline ALC, and their immune system’s individual response to radiation. Therefore, we also assessed lymphocyte count changes relative to the baseline, according to six levels (defined in Section 2.3). The cross-categorization between ALC grades at nadir and the change in ALC by the end of treatment, relative to the baseline, can be seen in **Figure 3**.

For groups A and B, categorization according to nadir ALC provides limited information, with the vast majority of both groups having no RIL. However, looking at the distribution amongst levels, we see a much more comprehensive and detailed picture. For example, from the 48 patients in group A that did not develop RIL as defined by ALC < 1000 K/μl, the ALC of 22 patients still declined by 5–25% (L1), and 14 patients in fact had an ALC increase of more than 5% from the baseline (L+). For group B, 50 patients did not reach the definition of RIL— yet 22 of these patients had an ALC decline of 25–50% (L2). In group C, the grades and levels are more agreeable in describing absolute lymphopenia severity and relative change in ALC.

#### 3.5.1 End of treatment sample

By the end of treatment, 44.2% of group A were in L1 (**Figure 3**). A notable 16 patients had an ALC increase by over 5% (L+). The rest of the patients were mainly in L0, with one patient reaching L2. There were no incidences of L3–L4. In group B, most patients were in L1 and L2 (81.7%), with four in L+, four in L0 and three in L3. No patients reached L4. For group C, a striking 73.9% of patients were in L3, showing a decline in ALC by 50–75% from the baseline. This majority was followed by 11 patients in L2, six reaching L4, one patient in L1, and no patients in L+ or L0, a marked contrast from groups A and B.

#### 3.5.2 First in-treatment sample

We observed a surprising rise in ALC amongst some patients at their first in-treatment sample. In group A, 44.2% had an increase of over 5% in ALC relative to the baseline. In groups B and C, 46.7% and 27.5% of patients, respectively, were also in L+, which was statistically significant (χ^2^ test; *p* = 0.039). The mean relative ALC increase for those in L+ was 24.4% (6.6–113.3%), 19.4% (5.4–42.7%) and 15.4% (5.6–64.2%) for groups A, B and C, respectively. L+ had the largest number of patients from groups A and B. For group C, most patients were in L1 during the sample’s period. By the second sample, the proportion of patients in L+ dropped to 30.8% in group A, 23% in group B, and 1% in group C. This was statistically significant for groups B and C (χ^2^ test; *p* < 0.001).

**Figure 4:**
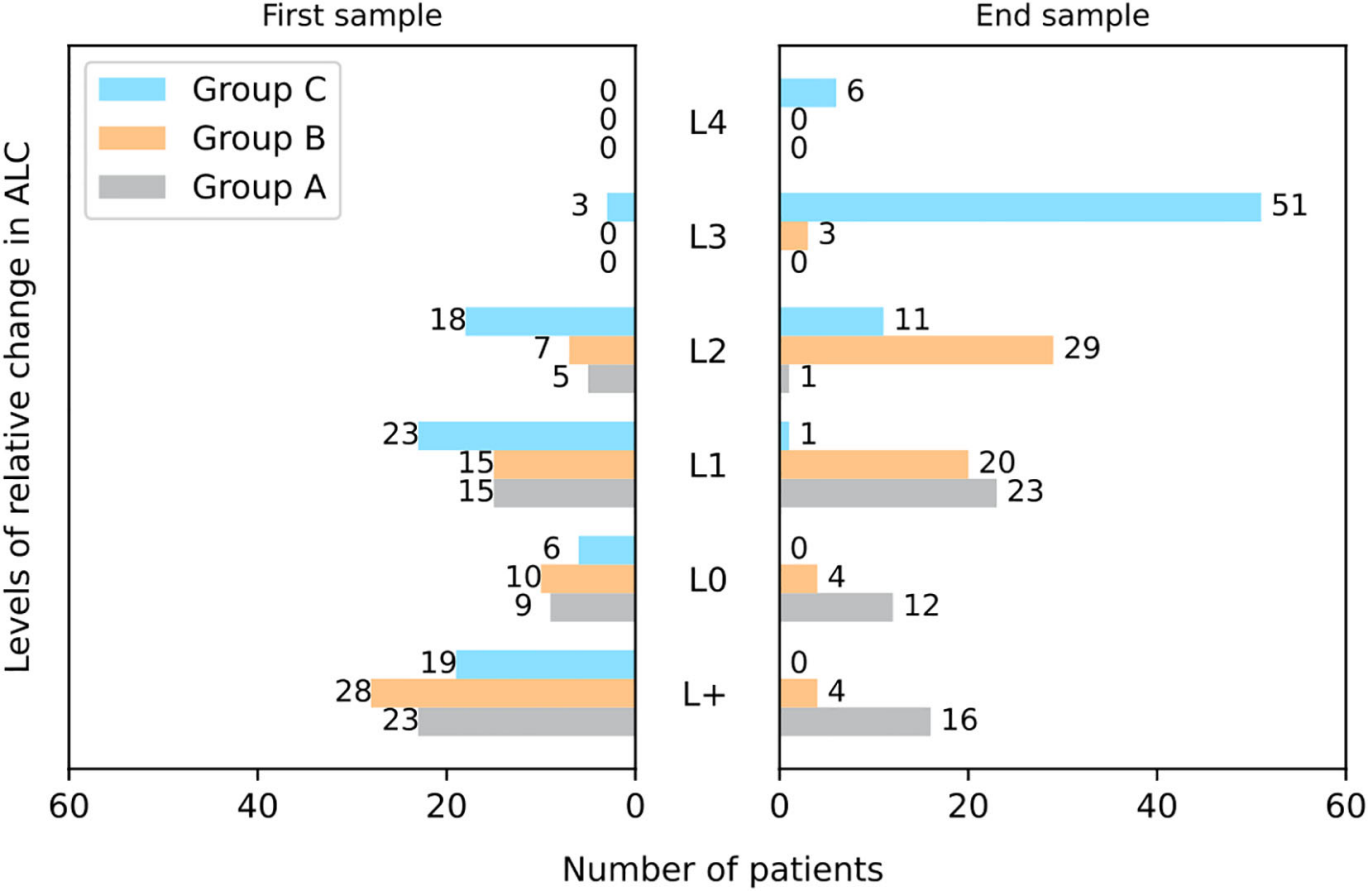
Bar charts showing the distribution of patients across lymphocyte count levels (L+ to L4) at the first week of treatment and at the end of treatment, for groups A, B and C. ALC thresholds defined as: L+ – ALC increase of more than 5% relative to the baseline, L0 – ALC increase of less than 5% and decrease of less than -5% relative to the baseline, L1 – ALC decrease between 5–25% relative to the baseline, L2 – ALC decrease between 25–50% relative to the baseline, L3 – ALC decrease between 50–75% relative to the baseline, L4 – ALC decrease of more than 75% relative to the baseline. ALC = absolute lymphocyte count.

Comparing the results of the first in-treatment sample to the end-of-treatment sample (**Figure 4**), we find no dramatic difference in level categorization for group A, but a substantial push towards higher levels of relative ALC decline in groups B and C. The number of patients in L+ goes from 28 to four and 19 to zero in groups B and C, respectively. For group B, L2 grows the most from seven to 29 patients. In group C, L3 increases substantially from three to 51 patients by the end of treatment.

To validate the statistical significance of the increase in ALC, we assessed patients from groups B and C that had their first in-treatment blood sample at a cumulative dose of exactly 6 Gy (Group B, n = 14; Group C, n = 20) or 9 Gy (Group B, n = 16; Group C, n = 13). Patients in L0 were excluded from further analysis. At 6 Gy, 50% of group B (6/12) and 59% (10/17) of group C were in L+ as opposed to L1-L4. The ratio was statistically significant (χ^2^ test; *p* = 0.001). For those with 9 Gy, 45.6% of group B (5/11) and 25% of group C (3/12) were in L+, which was also statistically significant (χ^2^ test; *p* =0.037).

### 3.6 Cumulative dose reached at nadir ALC

**Figure 5:**
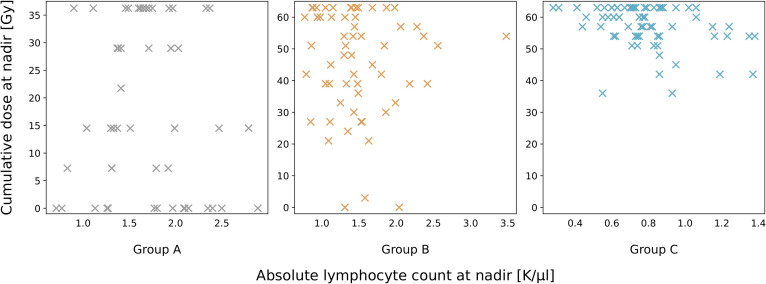
Scatter plots illustrating the relationship between the cumulative radiation dose delivered at the absolute lymphocyte count nadir, for groups A, B and C. Each point represents an individual patient, with the nadir dose corresponding to the lowest recorded lymphocyte count.

Reaching the nadir ALC is a pivotal moment, as it often corresponds to the height of radiation-induced lymphocyte depletion. Determining the dose at which the nadir occurs can provide valuable insight into patient responses to radiation. It is of course, individual for each patient, but distinct patterns emerge among each patient group (**Figure 5**). For group A, we see no discernible trend between the nadir ALC and cumulative dose. This is expected, as most patients did not develop RIL. The median nadir was 1.67 K/μl (range: 0.71-2.89 K/μl). For groups B and C, a distinct shift towards higher cumulative doses causing the nadir ALC begins to appear. In group B, the median nadir was 1.41 K/μl (range: 0.77-3.48 K/μl), occurring at a cumulative dose of over 20 Gy for almost all patients, save for some outliers. The nadir for all patients in group C was below 1.40 K/μl (median: 0.75 K/μl, range: 0.28-1.38 K/μl), arising after a cumulative dose of 30 Gy to high-dose targets, corresponding to the 10^th^ fraction.

### 3.7 Univariate and multivariate analysis results

A combination of treatment and clinical characteristics that passed the univariate significance criteria were evaluated using multivariate analysis. Assessing groups B and C (n = 129), baseline ALC, ISUP grade, initial PSA, DM, hypertension, hormonal therapy, PNI, dose at first sample, nadir, and end-of-treatment were found to be significant in the univariate analysis, and were subsequently included in the multivariate analysis. Results for the UVA can be found in **Supplementary Table S3** in the **Supplementary Material**. The results of the multivariate analysis are in **Table 2**. The outcome variable ALC < 500 K/μl was excluded as no patients from group B developed G3-4 RIL, so there would not have been any comparative value. Baseline ALC and PNI were deemed statistically significant for all outcome variables (*p* ≤ 0.05). The dose at first sample was statistically significant for the relative ALC change in the first week (*p* ≤ 0.05). The dose at nadir was statistically significant for the outcome ALC < 1,000 K/μl (*p* ≤ 0.05), and the dose at the end of treatment was significant for the relative change of ALC by the end of treatment (*p* ≤ 0.05).

**Table 2:** Results for multivariate linear regression (n = 129).

Variable	Relative ALC change in first week of treatment (β- coefficient, 95% CI, p-value)	Relative ALC change at end of treatment (β-coefficient, 95% CI, p-value)	ALC <1000 K/μl (β-coefficient, 95% CI, p-value)
***Baseline ALC***	0.69 (0.58–0.80), **<0.001**	0.43 (0.35–0.50), **<0.001**	-0.25 (-0.34–0.16), <**0.001**
***Age***	—	—	-0.004 (-0.01–0.01), 0.42
***ISUP Grade***	—	-0.005 (-0.05–0.04), 0.85	0.04 (-0.01–0.10), 0.15
***PSA (ng/mL)***	—	0.003 (-0.002–0.007), 0.24	-0.0007 (-0.01–0.004), 0.76
***Diabetes Mellitus (Yes/No)***	-0.08 (-0.26–0.11), 0.42	-0.11 (-0.24–0.02), 0.11	—
***Hypertension (Yes/No)***	—	0.06 (-0.06–0.18), 0.35	—
***Hormonal Therapy (Yes/No)***	-0.05 (-0.27–0.16), 0.62	0.11 (-0.03–0.26), 0.13	-0.03 (-0.20–0.15), 0.76
***Pelvic Node Irradiation (Yes/No)***	-0.22 (-0.43–0.01), **0.04**	-0.81 (-0.97–0.65), **<0.001**	0.55 (0.35–0.75), **<0.001**
***First Sample Dose (Gy)***	-0.04 (-0.06–0.02), **<0.001**	—	—
***Nadir Dose (Gy)***	—	—	0.006 (0.001–0.01), **0.01**
***End-of-Treatment Dose (Gy)***	—	-0.02 (-0.04–0.006), **0.007**	—

The 95% confidence interval (CI) provides the range within which the true effect size is likely to fall with 95% confidence. A p-value reflects the probability of the observed result occurring by chance, with values less than 0.05 considered statistically significant (highlighted in green, bold). Variables that were not significant in the univariate analysis for a given outcome were not included in the multivariate analysis, and therefore no values are provided for them. ALC, absolute lymphocyte count; DM, diabetes mellitus; HT, hypertension; PNI, pelvic node irradiation. The beta coefficient (β) represents the strength and direction of the association, with positive values indicating a direct relationship and negative values indicating an inverse relationship.

## 4 Discussion

It is important to observe the complete range of radiation-induced lymphocyte alterations, especially in patients who might experience significant shifts in lymphocyte levels but do not conform to the criteria of lymphopenia. This provides a more personalized understanding of individual responses to radiation therapy. Furthermore, this addresses a gap in current research by emphasizing the importance of both relative changes and absolute lymphocyte counts. For instance, we observed that some patients who did not meet the threshold of ALC < 1000 K/μL still experienced substantial alterations in their lymphocyte counts, with a large proportion having an ALC reduction of 25-50% (L2). In this study, classifying patients based on different levels of lymphocyte count changes has provided valuable insights into the proportion of patients likely to respond in a certain way, aiding in understanding the variability in individual reactions.

A patient’s baseline lymphocyte count should not be overlooked when evaluating radiation-induced changes in the ALC. The baseline ALC was a predictive factor (*p* ≤ 0.05) for both developing RIL, and percentage decline, in our MVA. Several other studies found baseline ALC or baseline lymphopenia to be predictive for developing RIL during radiotherapy for pelvic malignancies (15, 16). A higher initial ALC may act as a safeguard against entering lymphopenia during radiotherapy. In a study on 121 prostate cancer patients, a baseline ALC below 1,830/μL was predictive of a higher risk of acute G3 RIL, while a baseline ALC ≤ 1,780/μL increased the risk of G2 late lymphopenia (4).

An interesting trend emerged in our data— almost half of patients with no lymph node irradiation, and over a third of patients with PNI had an increase of over 5% in ALC relative to the baseline during the first in-treatment sample. Mohan et al. reported a similar increase in ALC relative to the baseline during the first week of RT in glioblastoma patients treated with proton or photon therapy, which was maintained into the second week before beginning to decrease (17). The mechanism leading to the observed increase, the subpopulation of lymphocytes responsible for it, and whether the increase is clinically relevant, remain unclear. Under specific conditions such as carefully selected doses, fractionation schedules, and target volumes that preserve the tumour microenvironment’s capacity to emit chemotactic and immunostimulatory signals and enable circulating immune cells to respond effectively, radiation may stimulate the immune system (7).

The effect of primed T-cells is not limited to the irradiated treatment volume. Case studies of metastases regressing after irradiation of the primary tumour have been published, and the phenomenon has been termed as the abscopal effect (18). This increase of tumour-specific lymphocytes is unfortunately hindered with continuous radiation. By the second in-treatment sample, the proportion of patients in L+ dropped substantially. This is also when the largest sample-to-sample decline for groups B and C occurred. At some point, the immunomodulatory effect of radiation switches to become predominantly immunosuppressive. John-Aryankalayil et al., who studied the gene expression profiles of prostate carcinoma cells, found the inflection point for increased immune-related transcripts to be at six to eight fractions of 1 Gy, with a maximum of 10 fractions (19).

Maintaining an adequate ALC is important not just for the body’s own anti-tumour response, but also for cases involving the use of immunotherapy. At present, there are several FDA-approved immunotherapies for prostate cancer, and more being tested (20). These are mostly used in castrate-resistant patients, a group that is growing in number (21). Such patients would benefit from a radiotherapy regime that takes their immune system into account, and perhaps is even used synergistically with immunotherapy. Radiotherapy could be utilised to stimulate and prime the immune system before boosting it with immunotherapy. In a prostate cancer transgenic mouse model, significant activation of anti-tumour T-cells was observed only when radiotherapy preceded immunotherapy by ideally 3–5 weeks in a combined treatment plan (22). A study by Altorki et al. on lung cancer patients reported that three consecutive daily fractions of 8 Gy stereotactic radiotherapy before the first cycle of durvalumab resulted in a major pathological response in 16/30 patients, in contrast to only 2/30 patients responding in the durvalumab monotherapy arm (23).

**Figure 6:**
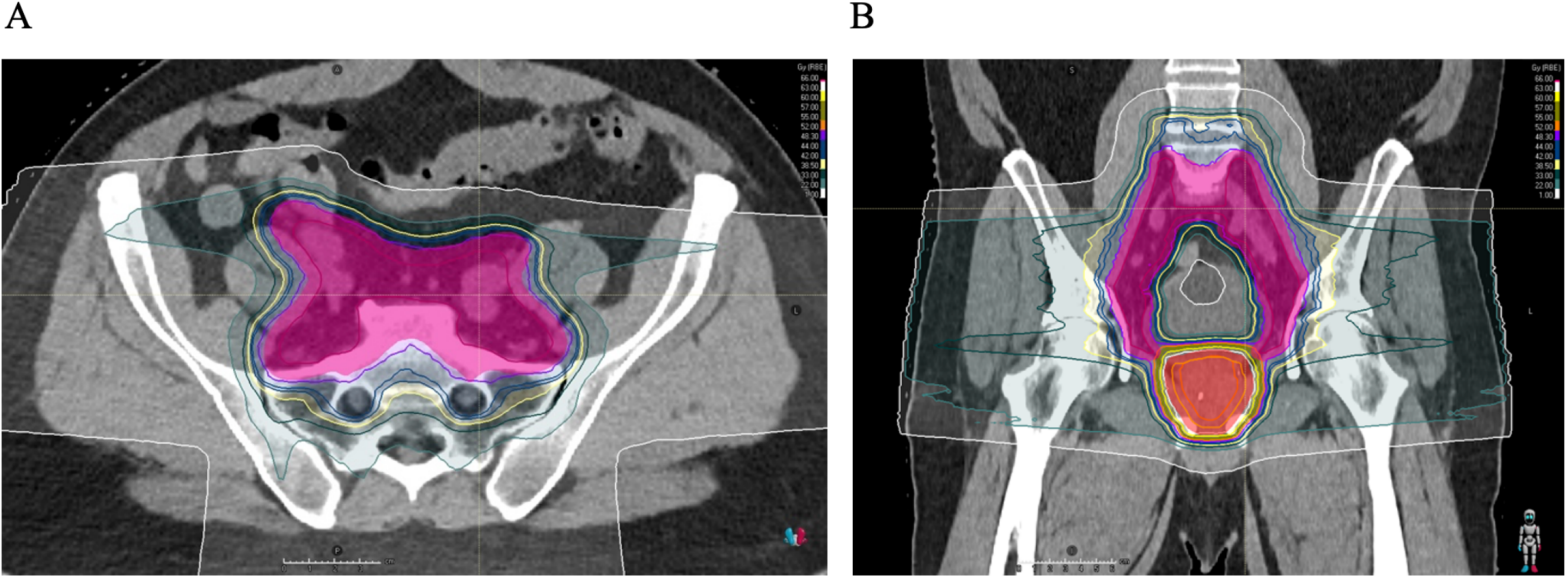
Transversal **(A)** and coronal **(B)** CT slices overlaid with a colourwash representation of the dose distribution for the treatment of prostate cancer with pelvic lymph node irradiation. High dose of 63.0 Gy to PTV of the prostate (shaded red), lower dose of 48.3 Gy to PTV of the lymph nodes and seminal vesicles (shaded pink, seminal vesicles not seen in these sections). Treatment planning done using the Raystation^®^ treatment planning system (RaySearch Laboratories AB).

In this study, patients who underwent pelvic lymph node irradiation had a markedly higher incidence of RIL than those who did not, and PNI was a statistically significant predictor for RIL in our multivariate analysis (*p* = 3.79e-07). The association between PNI and lymphopenia development during prostate radiotherapy has been reported in photon-based studies (4, 15, 24–26). Pelvic lymph nodes are irradiated because of the assumption of possible micro-metastasis to them. Elective PNI is frequently done in high-risk cancer patients, but the necessity for it in some cases is disputed, as questions remain about its place in the context of lymphopenia and subsequent potential reduction of overall survival (27). Irradiating the lymph nodes means a higher total planning treatment volume (PTV), which also leads to increased, unwanted radiation to the bone marrow (BM) and circulating lymphocytes, both of which have been shown to partake in the development of lymphopenia (15). An example of the PTV contouration for a prostate cancer patient undergoing PNI can be found in **Figure 6**.

The role of tumour-draining lymph nodes (TDLN) is pivotal in the body’s own anti-tumour immune response— which is why persistent tumours have evolved their own methods of evading the immune system, such as by releasing immunomodulatory exosomes and soluble mediators, creating an immunosuppressive environment that supports tumour growth (28). TDLN serve as the initial location where T-cells are primed against tumour antigens. Dendritic cells bearing tumour antigens travel to the TDLN, present them, and initiate activation of the antigen-naïve T-cells (29).

Progenitor cells of lymphocytes are produced in the bone marrow (30), however the extent to which the BM plays a role in replenishing lymphocytes during RIL remains unclear. 70% of the body’s BM is found in the pelvic bones and lumbosacral vertebrae, with up to half of all active BM in adults found in the region (31–34). Results from the INTERTECC-3 trial, which used PET-bone marrow-sparing IMRT, concluded that sparing the BM significantly reduced acute grade ≥ 3 neutropenia, but had no effect on RIL (35). Sini et al. reported that only a high dose of 40 Gy to the BM correlated with acute and late lymphopenia (4). An in-silico study by Baré et al. demonstrated that including bone marrow as an organ at risk with dose constraints was feasible without compromising dose targets or constraints (36). This approach led to a statistically significant reduction in the effective dose to circulating immune cells (EDIC), with respective decreases of 6.7% and 7.6% in EDIC values for patients receiving RT to the prostate or prostate bed, respectively, with pelvic node irradiation. These findings suggest that a straightforward strategy to mitigate lymphocyte dose in prostate cancer patients may be to incorporate pelvic bone marrow as an organ at risk.

The replenishment of lost lymphocytes is done by two distinct pathways— thymic maturation of precursor cells made by the BM, generally as a response to moderate/physiological lymphopenia, and peripheral homeostatic proliferation in the lymph nodes and spleen, in response to severe lymphopenia (37, 38). The latter may be more relevant in RIL, and although the process is age-independent, it becomes particularly important in older age, such as in our patient cohort, when there is increased involution of the thymus (37, 39). The majority of patients in Group C underwent hormonal therapy. It has been suggested that this treatment may ‘rejuvenate’ the thymus and increase thymic output in prostate cancer patients (40). Although hormonal therapy passed the univariate analysis in our study, it was not statistically significant in the multivariate analysis for influencing the relative change in ALC during the first week or at the end of treatment.

Lymphocytes circulating through the pelvic blood vessels and lymphatics are also included within the PTV when a patient undergoes PNI (41). Circulating lymphocytes are reported to be more radiosensitive than non-circulating lymphocytes found in the parenchyma or in the tumour (42). In a glioblastoma study using a 60 Gy/30-fraction treatment plan, 5% of circulating cells received 0.5 Gy/fraction, and > 95% of lymphocytes were exposed to 0.5 Gy over the treatment course (43). The occurrence of RIL during intra-cranial tumour irradiation is a push towards the idea that RIL can occur even in the absence of lymph node irradiation, when in areas with heavy blood flow.

This study used proton radiotherapy (PT) to assess its impact on lymphocytes, marking the first investigation into lymphocyte count changes induced by PT in patients with prostate cancer. Previous research investigated acute RIL in prostate cancer patients undergoing photon RT. One study reported G1+ RIL in all 121 patients treated with PNI, G2 in 61% and G3 in 25% (4). Cozzarini et al. used a variety of IMRT techniques and reported similar findings of G1+ in all 125 patients, G2 in 87% and G3 in 26% (26). The incidence of all grades of lymphopenia was lower in this study using protons, most notably for high-grade RIL (ALC < 500 k/uL).

Comparisons between proton and photon radiotherapy in other tumour sites consistently indicate that protons cause less RIL. In a study done on oesophageal patients, 40.4% of 136 patients developed G4 RIL with IMRT, versus 17.5% of 136 patients treated with protons (44). Researchers investigating glioblastoma patients found 39% of their 56 XRT-treated patients developed G3+ RIL, vs 14% of 28 PT patients (17). This is not surprising, due to the physical properties of protons and the occurrence of the Bragg peak, which allows for a lower entrance dose and sharp dose decline beyond the target volume (45). Consequently, the surrounding organs at risk are exposed to a lower dose compared to conventional photon radiotherapy. De Ornelas et al. conducted a dosimetric comparison between photon (VMAT) and proton (IMPT) plans, incorporating the pelvic bone marrow and its subvolumes as organs-at-risk to be spared. They concluded that IMPT was overall better at reducing mean and low doses to the BM, resulting in lower calculated rates of hematological toxicity (46). Particle-based therapies have not only dosimetric advantages, but potential immunomodulatory effects as well. In a study comparing photon and proton radiation on various tumour cell lines, proton-irradiated cells showed increased calreticulin cell-surface expression, a protein that enhances tumour cell sensitivity to cytotoxic T-cell killing (45).

In terms of minimising exposure to lymphocyte-rich organs at risk, the advantages might not be as pronounced in the pelvis. Current guidelines do not take radiation-induced immune changes into consideration, and neither pelvic blood vessels nor the pelvic bone are classified as organs at risk with specified dose constraints (47). However, protons could offer substantial benefits in cases involving lymphocyte-rich organs with established constraints, such as the heart or lungs. Should there be a shift towards optimizing treatments to reduce RIL and sparing pelvic lymphatic tissues, proton therapy could be a prime choice (1, 48–51).

Ultra-hypofractionated radiotherapy has recently become the standard of care for low and intermediate-grade prostate cancer (52, 53). The ultra-hypofractionated subgroup had a notably smaller incidence of any grade of lymphopenia compared to the subgroups with 21 fractions. Other studies have reported similar findings, suggesting that less fractions may conserve circulating lymphocytes (54). Furthermore, acute antigen release from tumours may be more effective at triggering an immune response than chronic release.

This research was limited by unavailable long-term follow up data to assess chronic lymphopenia, and to correlate the results with over-all survival, as well as by any potential bias that may come from a single-centre study. Patients with PNI had a higher incidence and worse grades of lymphopenia, but we could not separate to what degree this was due to radiation of the lymph nodes, BM or circulating lymphocytes, as they were not individually contoured. To the best of our knowledge, this is the first study investigating the effects of proton radiotherapy-induced changes in the lymphocyte count in prostate cancer patients with varying treatment plans. A multi-arm study assessing the differential impacts of photon versus proton therapy on RIL in prostate patients would be beneficial. We developed a categorisation for the lymphocyte count change relative to the baseline, which we believe paves the way to a more nuanced picture of the effect of radiation on individuals. Understanding the response of lymphocytes to varying doses, treatment volumes and node irradiation may offer opportunities for treatment optimization, ultimately improving patient outcomes.

## 5 Conclusion

The results of this investigation indicate that prostate cancer patients treated with proton radiotherapy exhibit varying levels of radiation-induced lymphopenia, with distinct dynamics of ALC changes, depending on the treatment approach. Patients who underwent pelvic node irradiation experienced a higher incidence of RIL compared to those who did not, with the majority of non-PNI patients showing no lymphopenia, while most PNI patients developed grade 2 lymphopenia. Baseline ALC and PNI were statistically significant predictors of both the development of lymphopenia and the relative change in ALC by the end of treatment compared to baseline. Furthermore, patients that had higher treatment doses experienced slightly higher rates of RIL, and those who underwent the ultra-hypofractionated regimen had the lowest incidence of RIL. These findings provide valuable insights into the factors influencing lymphocyte dynamics during proton therapy, contributing to a better understanding of how treatment parameters impact immune function.

## Data Availability

The raw data supporting the conclusions of this article will be made available by the authors, available upon request.
