# Supplementary material for: Temporal dynamics of lymphocytes in prostate cancer patients treated with proton therapy
Source: Front Oncol. 2025 Apr 16;15:1470876. doi: 10.3389/fonc.2025.1470876 (PMC12040820; doi:10.3389/fonc.2025.1470876)
Supplement: Supplementary file 1 [file Table1.docx]

Supplementary material

| **Factor** | **No pelvic node irradiation** | | | | | **Pelvic node irradiation** | | | |
| --- | --- | --- | --- | --- | --- | --- | --- | --- | --- |
| Dose received (Gy) | 36.25 | | 63.00 | | | | | 63.00 | |
|  | N/median | Range | | N/median | Range | | N/median | | Range |
| Number of Patients | 52 | - | | 60 | - | | 69 | | - |
| Age | 66.5 | 45–80 | | 70 | 49–79 | | 73 | | 53–84 |
| Adenocarcinoma | 52 | - | | 60 | - | | 69 | | - |
| T stage |  |  | |  |  | |  | |  |
| cT1(a-c) | 7 | - | | 14 | - | | 3 | | - |
| cT2(a-c) | 45 | - | | 46 | - | | 38 | | - |
| cT3(a-c) | 0 | - | | 0 | - | | 26 | | - |
| cT4(a-c) | 0 | - | | 0 | - | | 2 | | - |
| cN0 | 52 | - | | 60 | - | | 53 | | - |
| cN1 | 0 | - | | 0 | - | | 16 | | - |
| ISUP grade |  |  | |  |  | |  | |  |
| 1 | 25 |  | | 34 |  | | 10 | |  |
| 2 | 20 |  | | 19 |  | | 13 | |  |
| 3 | 7 |  | | 7 |  | | 11 | |  |
| 4 | 0 |  | | 0 |  | | 24 | |  |
| 5 | 0 |  | | 0 |  | | 11 | |  |
| Initial PSA, ng/mL | 6.5 | 2.0–19.5 | | 8 .0 | 1.6–14.9 | | 12.5 | | 2.8–93.6 |
| Hypertension | 24 | - | | 9 | - | | 23 | | - |
| Diabetes Mellitus | 8 | - | | 8 | - | | 17 | | - |
| Hormonal Therapy | 8 | - | | 10 | - | | 64 | |  |
| Baseline lymphocyte count | 2.0 | 0.71–3.33 | | 2.0 | 0.96–4.74 | | 2.0 | | 0.85–3.46 |
| Total PTV (cm^3^) | 118.4 | 67.3– 180.2 | | 171.6 | 50.1–355.4 | | 868 | | 595–1599.8 |
| Duration of RT (days) | 10 | 9–13 | | 29 | 28–33 | | 29 | | 28-34 |

Table S1: Patient characteristics.

|  | Sample 1 | Sample 2 | Sample 3 | Sample 4 |
| --- | --- | --- | --- | --- |
| **Days in treatment** | | | | |
| Group A | 2.81 ± 1.7 | 10.48 ± 1.11 |  |  |
| Group B | 3.58 ± 2.23 | 10.73 ± 2.36 | 17.78 ± 2.59 | 27.67 ± 2.07 |
| Group C | 4.19 ± 2.53 | 11.09 ± 2.64 | 18.39 ± 2.86 | 28.77 ± 1.89 |
| **Cumulative number of fractions** | | | | |
| Group A | 1.67 ± 0.55 | 4.62 ± 0.49 |  |  |
| Group B | 3.12 ± 1.33 | 7.95 ± 1.44 | 12.87 ± 1.73 | 19.77 ± 1.14 |
| Group C | 3.49 ± 1.5 | 8.22 ± 1.74 | 13.06 ± 1.81 | 19.33 ± 1.06 |
| **Cumulative dose** | | | | |
| Group A | 12.13 ± 3.99 | 33.46 ± 3.56 |  |  |
| Group B | 9.35 ± 3.99 | 23.85 ± 4.33 | 38.6 ± 5.2 | 59.3 ± 3.42 |
| Group C |  |  |  |  |
| Group C – HD | 10.48 ± 4.5 | 24.65 ± 5.22 | 39.17 ± 5.2 | 59.78 ± 3.18 |
| Group C – LD | 8.03 ± 3.45 | 18.9 ± 4 | 30.03 ± 4.17 | 45.83 ± 2.44 |

Table S2: Average and standard deviation for days in treatment, cumulative number of fractions and cumulative dose for in-treatment samples for groups A, B and C. HD = high dose, LD = low dose

| **Outcome** | Baseline ALC | Age | ISUP grade | PSA | DM | HT | Hormonal Therapy | PNI | First sample dose | Nadir dose | End-of-treatment dose |
| --- | --- | --- | --- | --- | --- | --- | --- | --- | --- | --- | --- |
| Rel. change at first week | 9.13e-05 | 0.70 | 0.25 | 0.25 | 0.10 | 0.36 | 4.77e-04 | 1.83e-03 | 3.71e-07 |  |  |
| Rel. change end-of-treatment | 4.03e-02 | 0.81 | 3.39e-09 | 0.01 | 0.03 | 0.07 | 6.70e-12 | 6.81e-27 |  |  | 2.91e-02 |
| ALC < 1,000 K/μl | 5.14e-05 | 0.20 | 7.71e-07 | 0.02 | 0.21 | 0.39 | 5.50e-08 | 2.32e-18 |  | 4.35e-07 |  |
| ALC < 500 K/μl |  | | | | | | | | | | |

Table S3: P-value results for univariate analysis. ALC = absolute lymphocyte count, DM = diabetes mellitus, HT = hypertension
